# Mailed self-sample HPV testing kits to improve cervical cancer screening in a safety net health system: protocol for a hybrid effectiveness-implementation randomized controlled trial

**DOI:** 10.1186/s13063-020-04790-5

**Published:** 2020-10-21

**Authors:** Jane R. Montealegre, Matthew L. Anderson, Susan G. Hilsenbeck, Elizabeth Y. Chiao, Scott B. Cantor, Susan L. Parker, Maria Daheri, Shaun Bulsara, Betsy Escobar, Ashish A. Deshmukh, Maria L. Jibaja-Weiss, Mohammed Zare, Michael E. Scheurer

**Affiliations:** 1 Center for Epidemiology and Population Health, Department of Pediatrics, Baylor College of Medicine, One Baylor Plaza, MS: 305, Houston, TX 77030 USA; 2 Dan L Duncan Comprehensive Cancer Center, Baylor College of Medicine, Houston, TX USA; 3 Department of Obstetrics and Gynecology, Morsani College of Medicine, University of South Florida, Tampa, FL USA; 4 Department of Medicine, Baylor College of Medicine, Houston, TX USA; 5 Department of Epidemiology, The University of Texas MD Anderson Cancer Center, Houston, TX USA; 6 Harris Health System, Houston, TX USA; 7 Department of Health Services Research, The University of Texas MD Anderson Cancer Center, Houston, TX USA; 8 Center for Health Services Research, Department of Management, Policy, and Community Health, The University of Texas School of Public Health, Houston, TX USA; 9 School of Health Professions, Baylor College of Medicine, Houston, TX USA; 10 Department of Family and Community Medicine, The University of Texas McGovern School of Medicine, Houston, TX USA

**Keywords:** Cervical cancer screening, Self-sample HPV testing, Patient navigation, Hybrid effectiveness-implementation designs, Hybrid type 2 designs, Pragmatic trials

## Abstract

**Background:**

Almost 20% of U.S. women remain at risk for cervical cancer due to their inability or unwillingness to participate in periodic clinic-based screening. Self-sampling has been shown to be an effective strategy for screening women for high-risk human papillomavirus (HR-HPV) infection in specific contexts. However, its effectiveness among medically underserved women in safety net health systems has not been evaluated. Furthermore, it is also unclear whether implementation strategies such as patient navigation can be used to improve the success of self-sample screening programs by addressing patient-level barriers to participation.

**Methods/design:**

The *Pr*ospective *E*valuation of *S*elf-*T*esting to *I*ncrease *S*creening (PRESTIS) trial is a hybrid type 2 effectiveness-implementation pragmatic randomized controlled trial of mailed self-sample HPV testing. The aim is to assess the effectiveness of mailed self-sample HPV testing kits to improve cervical cancer screening participation among patients in a safety net health system who are overdue for clinic-based screening, while simultaneously assessing patient navigation as an implementation strategy. Its setting is a large, urban safety net health system that serves a predominantly racial/ethnic minority patient population. The trial targets recruitment of 2268 participants randomized to telephone recall (enhanced usual care, *n* = 756), telephone recall with mailed self-sample HPV testing kit (intervention, *n* = 756), or telephone recall with mailed self-sample HPV testing kit and patient navigation (intervention + implementation strategy, *n* = 756). The primary effectiveness outcome is completion of primary screening, defined as completion and return of mailed self-sample kit or completion of a clinic-based Pap test. Secondary effectiveness outcomes are predictors of screening and attendance for clinical follow-up among women with a positive screening test. Implementation outcomes are reach, acceptability, fidelity, adaptations, and cost-effectiveness.

**Discussion:**

Hybrid designs are needed to evaluate the clinical effectiveness of self-sample HPV testing in specific populations and settings, while incorporating and evaluating methods to optimize its real-world implementation. The current manuscript describes the rationale and design of a hybrid type 2 trial of self-sample HPV testing in a safety net health system. Trial findings are expected to provide meaningful data to inform screening strategies to ultimately realize the global goal of eliminating cervical cancer.

**Trial registration:**

ClinicalTrials.gov NCT03898167. Registered on 01 April 2019.

**Trial status:**

Study start data: February 13, 2020. Recruitment status: Enrolling by invitation. Estimated primary completion date: February 15, 2023. Estimated study completion date: May 31, 2024. Protocol version 1.6 (February 25, 2020).

## Contributions to the literature

- The PRESTIS trial is a hybrid type 2 pragmatic randomized controlled trial that will assess the effectiveness of mailed self-sample HPV testing kits to improve cervical cancer screening participation among underscreened patients in a safety net health system, while simultaneously assessing patient navigation as an implementation strategy.
- Upon completion, PRESTIS will provide much-needed insight into the utility of self-sample HPV testing as a means to screen medically underserved women otherwise do not or under-attend for standard of care clinic-based screening.
- Although with methodological trade-offs, hybrid designs can advance knowledge of the clinical effectiveness of mailed self-sample HPV testing while establishing best practices to optimize implementation in real-world settings.

## Introduction

### Background and rationale

The implementation of clinic-based Papanicolaou (Pap) test screening for cervical cancer has dramatically reduced the incidence of the disease in the United States (U.S.) and other countries with widespread screening programs [1]. However, despite the $5.4 billion in costs incurred by these programs in the U.S. each year [2], as many as 20% of U.S. women remain at risk for cervical cancer as a result of screening non-attendance [3] (i.e., their inability or unwillingness to periodically attend for clinic-based screening according to national screening guidelines). Recent data indicate increasing rates of screening non-attendance among U.S. women [4]. These trends are particularly concerning given that over half of the 13,000 cases of invasive cervical carcinoma diagnosed in the U.S. each year [5] are diagnosed in un- or under-screened women [6, 7]. Cervical cancer results in over 4000 deaths annually, and its treatment and follow-up costs total over $440 million [2].

While screening non-attendance is largely driven by inadequate access to preventive care [3], multiple personal and cultural barriers also affect women’s participation in timely screening. These barriers include language and cultural differences with providers, discomfort during a pelvic exam, education/literacy, and health beliefs [8–10]. Many of these factors continue to adversely impact screening participation even after medically underserved women gain regular access to preventive care [10, 11]. The availability of licensed vaccines to prevent infection with the etiologic agent, high-risk human papillomavirus (HR-HPV), creates new opportunities to reduce the incidence of both pre-invasive and invasive cervical disease. However, persistently low rates of vaccine uptake mean that many U.S women will continue to face significant risk of developing a cervical cancer for at least the next several generations [12]. Under such circumstances, cost-effective strategies to improve existing screening programs will be required for the foreseeable future.

Testing self-collected cervicovaginal samples for HR-HPV has been shown to be an effective strategy to overcome multiple barriers that often hinder clinic-based screening [13–23]. Clinical platforms capable of identifying clinically significant HR-HPV infections at the time a woman undergoes a Pap test are currently a standard of care option that improve both the sensitivity and specificity of cervical screening [24]. Recently, HR-HPV testing alone was recommended by the U.S. Preventive Services Task Force as a tool for cervical cancer screening [25]. Substantial evidence indicates that samples can be collected by providers or by women themselves [26, 27], paving the way for the expansion of primary screening from clinical settings into women’s homes through mailed self-sample HPV testing kits. Mailed self-sample HPV testing has been evaluated in multiple trials, particularly in international settings, where it has been used as an additive strategy to increase primary screening participation among screening non-attendees of organized population-based screening programs [13–23]. To our knowledge, in the U.S., the HOMES study in the Kaiser Northwest System has been the only published trial to evaluate mailed self-sample HPV testing in the analogous setting of an integrated health system [28]. However, to date, no randomized controlled trials have been conducted in safety net healthcare systems or in healthcare systems with a predominantly racially/ethnically minority patient population. Evaluating mailed self-sample HPV testing in this context is important both because safety net health systems (i.e., those that offer access to care regardless of the patient’s ability to pay [29, 30]) serve a large proportion of socioeconomically disadvantaged individuals in the U.S. [30] and because racial/ethnic minorities, particularly Hispanic and non-Hispanic black women, shoulder a disproportionate burden of cervical disease [31–33].

In safety net health system settings, patients may face barriers that hinder the use of mailed self-sample HPV testing kits, including language barriers, low literacy, and distrust of the health system. Patient navigation is a patient-centered service delivery intervention designed to promote access to timely screening, diagnosis, and treatment of cancer and chronic diseases [34] by addressing barriers to care (i.e., access and financial barriers, communication and informational barriers; fear, distrust, and emotional barriers, and structural medical system barriers) [34]. Patient navigation has successfully been used to increase participation in cancer screening among racial/ethnic minorities and medically underserved populations [35]. In the context of a mailed self-sample HPV testing intervention, patient navigation may synergistically increase screening participation by providing the opportunity to motivate women and address knowledge gaps and personal and cultural barriers to screening [36]. Thus, patient navigation may be an effective implementation strategy to increase reach, adoption, acceptability, and use of mailed kits.

### Use of hybrid effectiveness intervention trials to optimize self-sample HPV testing

**Fig. 1:**
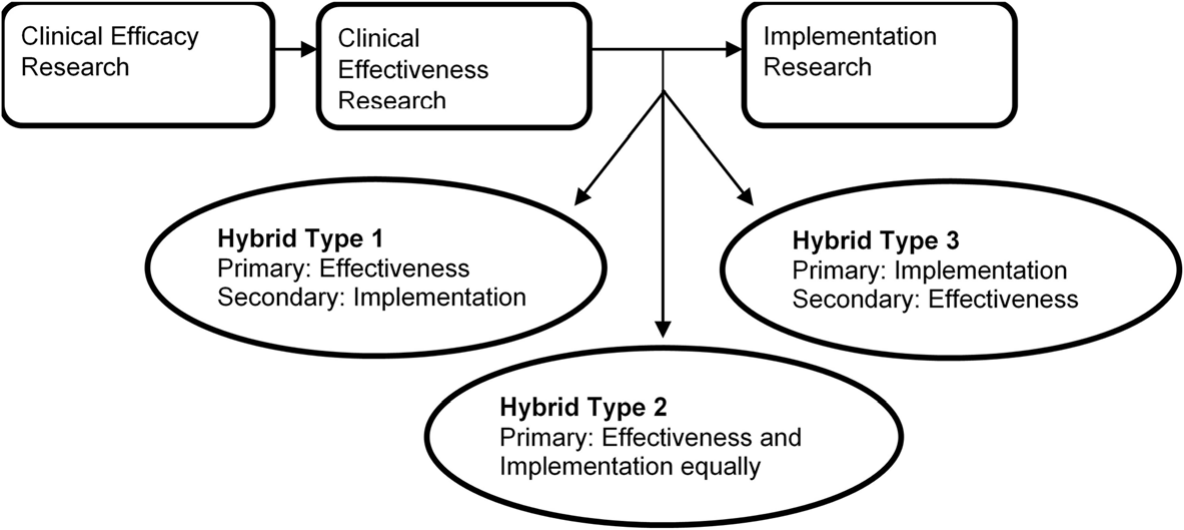
Hybrid effectiveness implementation trials

Hybrid effectiveness-implementation designs are research designs that address clinical effectiveness while incorporating and evaluating methods and procedures to implement interventions in real-world settings [37]. Their dual focus implies that hybrid studies may have a more rapid influence on clinical practice than standard clinical studies [38]. The continuum of hybrid effectiveness-implementation designs (shown in Fig. 1) range from those that emphasize effectiveness research with minimal implementation strategies (type 1) to those where effectiveness and implementation are given equal weight (type 2) to those that focus primarily on implementation outcomes with minimal focus on clinical effectiveness (type 3) [37, 39]. Hybrid type 2 designs are ideal when studying interventions that have demonstrated effectiveness in other settings or populations, but where there is uncertainty about the effectiveness of the intervention in another context [39, 40]. Type 2 designs are also ideal when there is momentum in a healthcare system to implement interventions with preliminary effectiveness data, as they provide an opportunity to study the intervention’s effectiveness while evaluating how best to implement the intervention [39]. As described by Landes et al., there are key criteria of type 2 designs to which such studies need to adhere. Specifically, it is critical in type 2 designs that an implementation strategy that is plausible in the real world be explicitly described, that implementation outcomes (e.g., reach, fidelity) be explicitly evaluated, and that there is a clear distinction between intervention components and intervention strategy components [39].

Here, we describe the rationale and design of the PRESTIS trial (*Pr*ospective *E*valuation of *S*elf-*T*esting to *I*ncrease *S*creening), a hybrid type 2 effectiveness-implementation pragmatic randomized controlled trial (RCT). The overarching goal of the PRESTIS trial is to simultaneously evaluate the effectiveness of mailed self-sample HPV testing kits to improve cervical cancer screening participation among patients overdue for clinic-based screening while evaluating patient navigation as a strategy for their implementation. The driving hypothesis is that mailed self-sample HPV testing kits and patient navigation act synergistically to increase cervical cancer screening in otherwise underscreened patients. Informed by the Reach, Effectiveness, Adoption, Implementation, Maintenance (RE-AIM) framework [41], PRESTIS also aims to determine implementation outcomes of mailed self-sample HPV testing; specifically, reach, patient acceptability, fidelity, adaptations, and cost-effectiveness.

## Methods

### Overview of trial

**Fig. 2:**
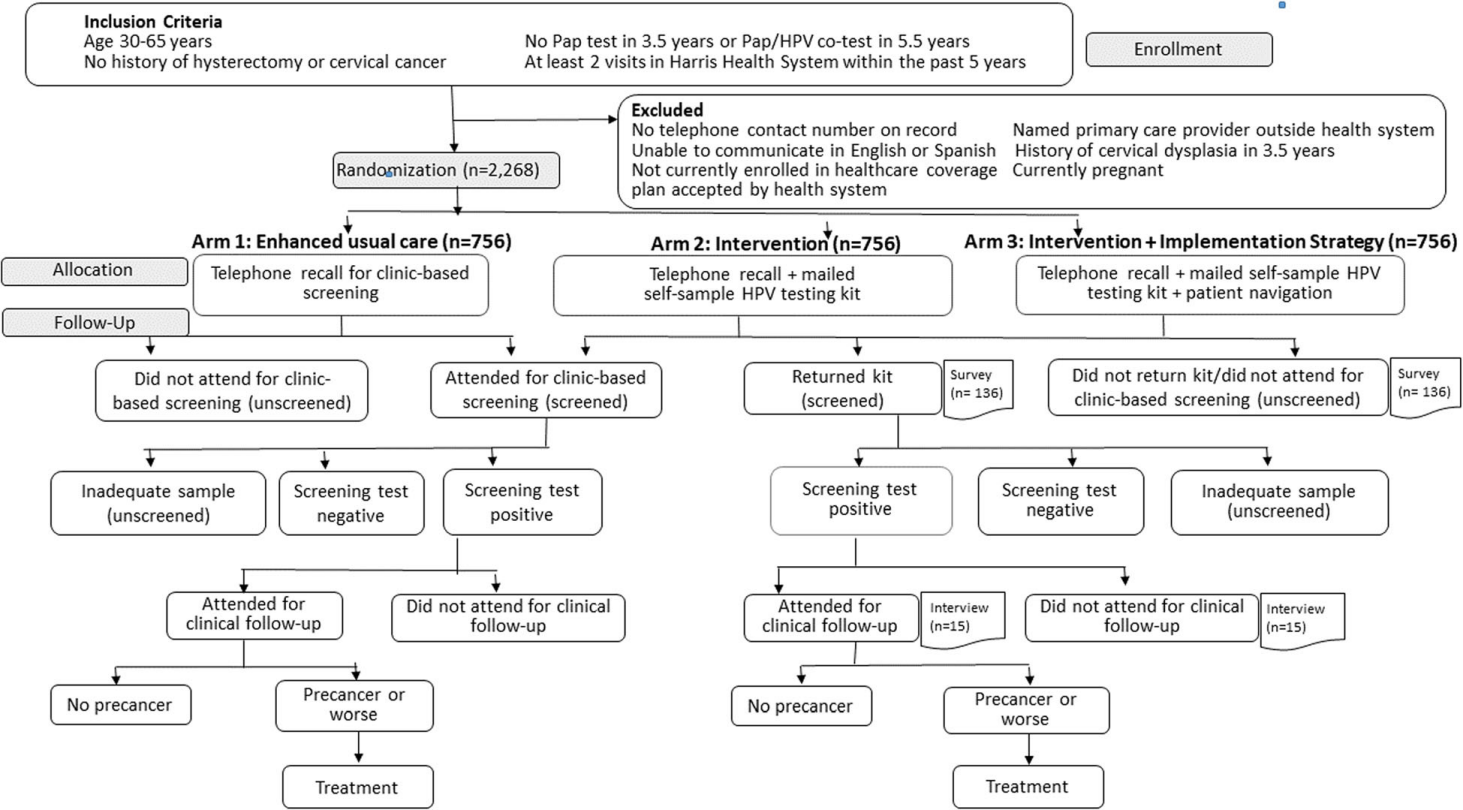
Design of the PRESTIS (*Pr*ospective *E*valuation of *S*elf-*T*esting to *I*ncrease *S*creening) trial

PRESTIS is a parallel, single-blinded, three-arm RCT (Fig. 2). The study arms are (1) telephone recall (enhanced usual care control arm), (2) telephone recall with mailed self-sample HPV testing kit (intervention arm), and (3) telephone recall with mailed self-sample HPV testing kit and patient navigation (intervention + implementation strategy arm). The primary effectiveness outcome is completion of primary screening, defined as completion and return of mailed self-sample kit or completion of a clinic-based Pap test. Secondary effectiveness outcomes are predictors of screening and attendance for clinical follow-up among women with a positive screening test and detection and treatment of cervical precancers (exploratory). Implementation outcomes are reach, acceptability, fidelity, adaptations, and cost-effectiveness.

### Conceptual framework

Multiple conceptual frameworks underlie the PRESTIS trial, including classic theories (i.e., PRECEDE-PROCEED) to develop the intervention, and implementation science frameworks (i.e., RE-AIM) to design and evaluate its implementation. PRESTIS is also informed by effectiveness-implementation hybrid design and pragmatic trial considerations.

The intervention was developed using the PRECEDE-PROCEED theoretical framework [42]. In our preliminary studies, the PRECEDE process was used to assess the individual, interpersonal, and environmental determinants of Pap test underutilization among women in our healthcare system. As described, there are multiple personal and cultural barriers that affect women’s participation in screening, in addition to structural barriers such as inadequate access to healthcare and lack of health insurance. Cultural and personal barriers include language and cultural differences with providers, discomfort during a pelvic exam, limited education/literacy, modesty concerns, unacceptability of a male provider, and lack of partner support for seeking screening. During the conception and planning of the intervention, we constructed a PRECEDE-PROCEED logic model that identified the predisposing, enabling, and reinforcing factors that could influence women’s decisions to participate in self-sample HPV testing. Key features of the study design were included to address these factors.

The implementation science conceptual framework underlying study design is the Reach, Effectiveness, Adoption, Implementation, Maintenance (RE-AIM) evaluation framework [41]. RE-AIM has been widely used to translate research into practice and plan for and evaluate the implementation of evidence-based programs in “real-world settings.” The PRESTIS trial is also informed by the pragmatic-explanatory continuum indicator summary 2 (PRECIS-2) pragmatic trial framework [43]. PRECIS-2 is a validated tool to guide trialists in making design decisions that are consistent with the trial’s intended purpose. It uses nine domains to describe clinical trials on a continuum from explanatory (ideal situation) to pragmatic (usual care situation): eligibility, recruitment, setting, delivery, adherence, follow-up, outcomes, and analysis [43]. Gold standards for pragmatic trials include broad eligibility criteria, an intent-to-treat data analytic approach, and incorporation of rigorous prospective controls.

### Protocol approvals, registration, and reporting guidelines

The trial was approved by the Institutional Review Board (IRB) for Baylor College of Medicine and granted administrative approval by Harris Health System. The trial is registered at ClinicalTrials.gov (NCT03898167). The SPIRIT checklist for RCT reporting is included in Additional file 1.

### Study setting

The PRESTIS trial is being conducted within Harris Health System in Harris County, Texas. Harris Health is the primary safety net provider for the Houston metroplex and provides care for more than 320,000 under- or uninsured Harris County residents. It is the third largest safety net health system in the U.S. and serves a racially and ethnically diverse patient population (predominantly Hispanic and non-Hispanic Black). Standard of care cervical cancer screening at Harris Health currently involves routine Pap test screening every three years for women 21 to 29 years and Pap and HR-HPV co-testing every five years for women 30 to 65 years. Pap and HR-HPV co-testing was adopted as standard of care for routine population-based cervicovaginal screening in 2015. Harris Health uses opportunistic strategies to promote screening, in particular, electronic medical record (EMR)-based identification of women due or past due for screening when they present for primary care and display of patient education videos in examination rooms [44]. Screening test results are managed according to the 2012 American Society for Colposcopy and Cervical Pathology (ASCCP) Consensus Guidelines [45] and 2015 ASCCP Interim Guidelines [46]. Women with an abnormal Pap test are tracked and navigated for clinical follow-up (i.e., colposcopy or repeat Pap test). Standard patient navigation procedures used at Harris Health System include educating and motivating patients to attend for follow-up, alerting providers to notify patients of their results and/or to perform necessary tests and procedures, directly booking Pap test appointments at primary care clinics, assisting patients in booking colposcopy appointments, and helping patients address other individual-level barriers (e.g., childcare and transportation needs).

### Integration of trial elements into health system

The trial was designed in collaboration with team members who serve as clinicians and leaders in the Harris Health System. The outreach interventions in each arm of the trial are delivered by trained Harris Health patient navigators who are embedded in a well-established and successful patient navigation program that has been successfully used to improve rates of cancer screening and diagnostic follow-up. Patient navigators participating in PRESTIS are well versed in standard procedures used at Harris Health System to identify and address barriers to care (as described above). In addition to standard patient navigation training, navigators have received research training on protection of human subjects, clinical trial database entry, and management. When contacting subjects on behalf of PRESTIS, the navigators deliver a scripted, arm-specific educational intervention. Self-sampling kits mailed to participants can be returned to Harris Health via U.S. Postal Service or by depositing them at well-identified laboratory drop-boxes at established Harris Health clinics. This latter strategy mimics the procedures used for home-base fecal immunochemical test (FIT) kits already used for population-based screening by many Harris Health patients. Kits are checked in by the patient navigation team and routed to the central health system laboratory for testing. Test results are posted as a research note in the EMR for clinical use by providers. Clinical follow-up for abnormal HR-HPV test results is coordinated by patient navigators and managed by Harris Health primary care and gynecology care providers. Trial information was disseminated to providers using existing communication pathways, including medical directors’ meetings, clinic staff meetings, and clinical update emails.

### Stakeholder Advisory Board

The trial’s Stakeholder Advisory Board (SAB) is comprised of representatives from Harris Health System leadership, ambulatory care providers, oncologists, patient navigators, and Harris Health patients. Responsibilities for the SAB include monitoring and guiding trial planning and execution, guiding the development of bilingual patient-centered education materials, providing input for the interpretation of study outcomes, planning for dissemination, troubleshooting to resolve scientific, logistical, and administrative challenges that may occur, and setting priorities for future research. In addition to semi-annual in-person and/or virtual meetings, the research team is in contact with SAB members by email for *ad hoc* issues.

### Eligibility and randomization

A waiver of informed consent was granted by the IRB to identify potentially eligible women through a query of the EMR. As determined by data abstracted from the EMR for each potential subject, women are eligible for PRESTIS if they are (1) aged 30–65 years, (2) have no history of prior hysterectomy or cervical cancer, (3) have had at least two visits within ambulatory care program of Harris Health System in the past five years, (4) have not had a Pap test in the past 3.5 years or a Pap/HPV co-test in the past 5.5 years, and (5) are currently enrolled in a healthcare coverage or financial assistance plan accepted by Harris Health (i.e., Harris Health financial assistance plan, Medicaid/Medicare, homeless grants, family planning grants, women’s health grants, Breast Cervical Cancer Screening (BCCS) grants, private insurance, and veterans’ health plans). A six-month grace period was added to the recommended screening intervals of three and five years for Pap and Pap/HPV co-testing, respectively, to allow time for women to respond to opportunistic usual care strategies.

Once initial qualifications are assessed, data of potentially eligible women are extracted from the EMR and transferred to REDCap electronic data capture tools hosted at Baylor College of Medicine [47, 48]. REDCap (Research Electronic Data Capture) is a secure, web-based software platform designed to support data capture for research studies, providing (1) an intuitive interface for validated data capture, (2) audit trails for tracking data manipulation and export procedures, (3) automated export procedures for seamless data downloads to common statistical packages, and (4) procedures for data integration and interoperability with external sources. Exclusion criteria are then assessed for potential participants based on a review of their EMR. Potential participants are excluded if they (1) lack telephone contact information in their EMR, (2) are not currently enrolled in a healthcare coverage plan accepted by Harris Health (re-assessed using financial assistance database and EMR), (3) have a named primary care provider outside the health system documented in their EMR (as this usually indicates referral to the health system for specialty care), or (4) have a history of cervical dysplasia in the past 3.5 years.

Women who meet the eligibility criteria are contacted by telephone. Participants who are not reached on first attempt are contacted on three different days at three different times before being classified as unreachable. At the time of the telephone call, additional exclusion criteria are assessed. Specifically, women are excluded if (1) they or a proxy are unable to communicate in English or Spanish and (2) report that they are currently pregnant. Once patient-reported exclusion criteria are assessed during the initial part of the phone call, a computer-generated randomization scheme in REDCap is used to randomly assign women who meet the eligibility criteria to the three trial arms. Patient navigators thus are not blinded to study arm assignment.

All eligible women are enrolled in the trial under a waiver of consent in order to reduce participation bias. A waiver of written documentation of informed consent was granted for participants’ use of the kits, due to the minimal risks involved and to enhance generalizability of the findings. A research information letter (described below) is used in lieu of a formal informed consent form.

### Study arms and interventions

Due to the transitory nature of our patient population, we decided that eligibility should be contingent on verification that participants can be reached by telephone and that they have a correct address on file. Thus, the control arm involves an initial telephone encounter to recall the participant to clinic-based screening rather than “usual care” which would rely only on opportunistic screening at clinics. The intervention arms incrementally add the intervention and implementation strategy (Table 1). The participant timeline is shown in Table 2.

**Table 1:** Arm-specific interventions and implementation strategies

Study arm	Interventions/implementation strategies^a^		
	Telephone recall	Mailed HPV self-sampling kit	Patient navigation
Arm 1 (enhanced usual care control)	X		
Arm 2 (intervention)	X	X	
Arm 3 (intervention + implementation strategy	X	X	X

^a^X indicates activities incorporated into each arm of the trial

**Table 2:** Participant timeline

Timepoint	Study period					
	Enrollment	Post-allocation				
	***Day 1***	***+ 2 days***	***+ 3–5 days***	***+ 6 months***	***+ 12 months***	***+ 18 months***
**Enrollment:**						
**EMR-based eligibility screen**	X					
**Telephone-based eligibility screen**	X					
**Allocation**	X					
**Interventions:**						
***Telephone recall***	X					
***Mailed HPV self-sampling kit***		X				
***Patient navigation***			X			
**Assessments:**						
***Socio-demographics***	X					
***Screening participation***				X		
***Screening test results***				X		
***Clinical follow-up***					X	
***Histology***					X	
***Treatment***						X

#### Telephone recall (all arms)

Participants receive a scripted telephone recall from a trained patient navigator. Patient navigators state that they are calling on behalf of Harris Health, inform participants that their records indicate that they are overdue for a Pap test, instruct them to call the scheduling department to make an appointment, and provide them the scheduling department’s telephone number.

#### Mailed HPV self-sampling kit (arms 2 and 3)

During the telephone recall encounter, patient navigators inform participants that they will receive a self-sampling kit in the mail. Patient navigators confirm the participants’ addresses and mail them a kit via U.S. Postal Service.

#### HPV self-sampling kits

Each HPV sampling kit consists of a commercially available cervical specimen collection kit (Aptima Cervical Specimen Collection and Transport Kit) along with instructions and materials that can be used to return completed kits. The Aptima kit consists of an individually wrapped cervical swab and a vial of Aptima Specimen Transport Medium. The kit is accompanied by a letter on behalf of health system medical leadership inviting potential subjects to participate in self-sample HPV testing as part of a research study; a brochure that instructs subjects on self-sampling and specimen collection; a research information sheet providing additional information about PRESTIS; and a pre-paid, return-addressed padded envelope. The introductory letter informs participants that national and local cervical cancer screening guidelines recommend cervicovaginal screening every three years or a combination of traditional cytology and HR-HPV co-testing every five years. The letter then indicates that, as an alternative to a Pap test, recipients can complete and return the enclosed self-sampling kit within four weeks. The additional research information sheet further describes the PRESTIS’s purpose, procedures, the voluntary nature of participation, potential risks and benefits, and protections for subject privacy and confidentiality. It indicates that use and return of the kit indicates consent to participate. It also provides the telephone number where subjects can call to schedule an appointment to be screened for cervical cancer at an established Harris Health primary care clinic if traditional screening if preferred by recipients. The instructional brochure that accompanies the test kit provides illustrated, step-by-step instructions in English and Spanish and is written for comprehension at a fourth-grade reading level.

#### Return of kits

The instructional brochure indicates that completed kits can be returned to Harris Health via the U.S. Postal Service using the pre-paid, return-addressed envelopes that accompany each kit or by physically returning the completed kit to a labeled laboratory drop box at an established Harris Health clinic. As mentioned, the latter option was included to mimic procedures used for mailed FIT kits for colorectal cancer screening that may already be familiar to some participants. It also reflects the observation that many patients opt to return their pre-paid, return-addressed FIT kit in person rather than by mail.

#### Reminder call

Participants who have not returned a kit within three weeks of the telephone recall receive up to three telephone reminders.

#### Laboratory testing for HR-HPV using self-sampling kits

Laboratory testing is conducted in the CLIA-certified central health system laboratory. HPV testing is conducted using the Aptima® HPV test (Hologic) per the manufacturer’s instructions on approved hardware compatible with this testing platform. Harris Health uses the Aptima testing platform for standard of care clinical HPV testing throughout its outpatient clinics. Aptima is an mRNA test that detects 14 high-risk HPV genotypes, including HPV-16 and HPV-18. Per local and national standard of care, HR-HPV-positive samples are reflexively tested specifically for HPV-16 and HPV-18/45, the genotypes associated with approximately 70% of invasive cervical cancers worldwide [49]. This two-tier strategy is used to improve risk stratification and determine the appropriate algorithm for subsequent clinical evaluation. Upon completion of HR-HPV testing, clinical results are interpreted as (1) HR-HPV negative, (2) HR-HPV positive and HPV 16/18/45 negative, (3) HR-HPV positive and HPV 16/18/45 positive, or (4) inadequate due to unsatisfactory sample. For the purposes of PRESTIS, sampling kits that are returned > 30 days after the mail date are categorized as clinically inadequate.

#### Management of test results

HR-HPV test results are reviewed by the study’s clinical co-investigator (EYC) prior to notification of results. Notification of results to participants is conducted by the patient navigators, who contact participants by telephone within 10 days of the laboratory’s receipt of the sample. Participants who are unreachable after three telephone attempts on different days/times are mailed their results by certified mail. Patient navigators document HR-HPV test results in the patients’ EMR under a research note and in the scanned media tab. Result notification and clinical follow-up procedures are as follows:

- *HR-HPV negative*. Participants with HR-HPV negative self-test results are informed that no high-risk HPV strains were detected in their sample. They are also informed that because HR-HPV self-sampling is not currently approved for primary screening, they should attend for clinic-based screening within the next 12 months.
- *HR-HPV positive (HR-HPV-16*, *HR-HPV-18/45 negative)*. Participants who test positive for HR-HPV but test negative for HR-HPV-16 or HR-HPV-18/45 are informed of their results and that additional clinical evaluation is recommended. While on the telephone with the patient, patient navigators are able to directly book appointments for clinic-based evaluation by Pap/HR-HPV co-testing at Harris Health primary care clinic locations.
- *HR-HPV-16, HR-HPV-18/45 positive*. Women who test positive for HR-HPV-16, HR-HPV-18/45 are informed of their results and that additional clinical evaluation is recommended. Patient navigators contact one of Harris Health's colposcopy clinics to request an appointment for colposcopy.
- *Clinically Inadequate Specimen*: Women whose samples are deemed clinically inadequate (due to unsatisfactory sampling or kit returned after > 30 days) are informed of the results and that they should attend for clinic-based screening. While on the telephone with the patient, patient navigators are able to directly book appointments for clinic-based screening at Harris Health primary care clinic locations.

#### Patient navigation (arm 3 only)

Within three to five days of the kit’s mail-out, participants in arm 3 receive a telephone call from a PRESTIS patient navigator. The primary purpose of this call is to provide one-on-one education in three overlapping domains: (1) information on the nature and purpose of cervical cancer screening and the causative role of HR-HPV, (2) the ability to complete screening through a clinic-based Pap test or through self-sample HPV testing using the kit, and (3) instruction on how to use and return the completed kit. This call also provides an opportunity for the patient navigator to answer questions and address concerns.

### Surveys and semi-structured interviews

Using a factorial design, a random sample of participants in arms 2 and 3 (self-sample HPV testing kit with patient navigation, who do and do not return their self-sampling kit) are invited to participate in a telephone survey. The survey is designed to identify attitudes and experiences toward cervical cancer screening, self-sampling, and patient navigation. Different versions of the questionnaire are used for kit returners and non-returners and for those who did/did not receive patient navigation. Additionally, a purposively selected subset of participants from arms 2 and 3 who test positive for HR-HPV (*n* = 30) will be invited to participate in semi-structured telephone interviews within six months of their test results. The telephone interview will assess women’s perspectives of an HR-HPV positive test and their experiences attending (or not attending) for clinical follow-up. Participation in the telephone survey and interviews requires verbal informed consent and participants are mailed a gift card incentive ($20 and $25 for survey and in-depth interview participants, respectively).

### Microcosting

To assess direct health-related and unrelated implementation costs necessary for evaluating cost-effectiveness, we are using a micro-costing approach that allows for precise estimation of economic costs associated with health intervention. In particular, we utilize a direct measure method of micro-costing that involves tracking resource use and enumeration of the unit costs of each of those resources to precisely estimate the cumulative costs associated with each intervention arm [50]. The objective of this assessment is to determine the costs of implementing each intervention, where we will consider cost components including training staff, identifying and contacting eligible women, and delivering the intervention. A cost tracking database is being used to itemize, quantify, and value self-sampling kit supplies, mailing costs, and laboratory testing.

### Outcomes measures

#### Clinical effectiveness outcomes

##### Primary outcome

The primary outcome is screening participation, defined as completion and return of a mailed self-sample HPV testing kit that is adequate for testing (i.e., does not produce unsatisfactory results) or attendance for clinic-based screening within six months of randomization. Screening participation, and other clinical effectiveness outcomes, will be ascertained by research coordinators who are not involved in the delivery of the intervention and who are blinded to arm allocation. Attendance for clinic-based screening and/or return of an adequate mailed self-sample HPV testing kit will be ascertained based on a review of the EMR. Participants in all arms who have no documented completion of a screening test within six months of randomization will be contacted by the patient navigators via telephone to assess whether clinic-based screening was performed elsewhere. Authorization of disclosure of medical information will be requested and, if granted, the outside provider will be contacted to validate the self-report. Primary screening participation is categorized as screened, unscreened, and lost-to-follow-up. Returned kits with inadequate samples are classified as unscreened; Pap tests done elsewhere are categorized as screened once confirmed by the outside provider; participants who we are unable to reach by telephone after five attempts are classified as lost-to-follow-up.

##### Secondary outcomes

*Secondary outcomes* include screening test results (positive, negative, or inadequate) and completion of clinical follow-up among women with an abnormal screening test result (attended, did not attend). Screening test results will be ascertained within six months of randomization, based on EMR review and laboratory results. Completion of clinical follow-up is ascertained by EMR review within six months of the date of the screening test result. Completion of clinical follow-up is defined as attendance for colposcopy among participants who had a positive test by clinic-based screening and attendance for colposcopy or subsequent clinic-based screening among those who had a positive test by self-sampling. Additional exploratory outcomes are the detection and treatment of cervical precancers (i.e., histologically confirmed cervical intraepithelial neoplasia grade II or greater [CIN2+]). Precancers will be ascertained by EMR review within six months of abnormal screening results. Treatment as per ASCCP guidelines will be ascertained within six months of the date of diagnosis.

#### Implementation outcomes

RE-AIM-informed implementation outcomes of the PRESTIS trial and their assessment methods are described in Table 3. Reach of the intervention will be measured through enrollment patterns in the recruitment log as well as patient flow through the protocol to assess the impact of specific eligibility criteria. Traditional RCT analyses, using an intent-to-screen approach, will be used to assess the effectiveness of the intervention and the implementation strategy. Sociodemographic, health, and healthcare utilization characteristics of women who complete primary screening will be described and compared across screening test modality to assess patterns of screening test utilization. Women’s experiences and attitudes toward self-sample HPV testing, follow-up of HR-HPV positive test results, and how they are mediated by patient navigation will be assessed using a survey and in-depth qualitative interviews among a subset of participants. Fidelity to the planned intervention, as well as adaptations made prior to and potentially during implementation, are being documented through detailed project notes and study timeline. The cost-effectiveness of the interventions (informed by costs and effectiveness data from the trial) will be evaluated using a disease microsimulation model. The model will first simulate cervical cancer natural history from the acquisition of HR-HPV infection to its potential persistence and progression to cervical cancer [51–53]. The outcomes of implementing the interventions will then be overlaid on the model to determine how longitudinal outcomes of these strategies (including lifetime costs, survival, and quality of life) vary at different levels of participation and willingness-to-pay thresholds.

**Table 3:** RE-AIM-informed implementation outcomes of the PRESTIS trial and their assessment methods

Assessment	Assessment method	RE-AIM domain
Patient flow through protocol	RCT data	Reach
Enrollment patterns	Recruitment log	Reach
Clinical effectiveness	RCT data, intent-to-treat analyses	Effectiveness
Characteristics of women who complete primary screening, by screening test modality	RCT data, intent-to-treat analyses	Adoption, implementation
Acceptability	Survey	Adoption, implementation
Experiences	Survey, in-depth interviews	Implementation
Fidelity/adaptations	Project notes	Implementation
Cost-effectiveness	Micro-costing, microsimulation model	Maintenance

### Data management and statistical analysis

#### Data management

Trial data are managed using REDCap and OnCore (Forte Research Systems, Madison, WI). Data of patients meeting the initial eligibility criteria are extracted from the EMR and transferred to REDCap for assessment of exclusion criteria initially based on EMR data, followed by the assessment of patient-reported exclusion criteria by telephone. Once exclusion criteria have been assessed, a computer-generated permutated block randomization scheme in REDCap is used to randomly assign women who meet the eligibility criteria to the three trial arms with a 1:1:1 ratio using participants’ medical record number. Randomized women are entered into OnCore and receive a study-specific identification number. Occurrence of participation-related events and procedures, as well as clinical outcomes, are recorded in OnCore. Electronic research data are stored in a secure, password-protected institutional server. Only study personnel and investigators have access to the data.

#### Statistical analysis

All primary and secondary outcome measures will be analyzed using an “intent-to-screen” approach. Bivariable tables and Pearson’s *χ*^2^ tests will be used to compare the proportion of outcomes by study arm, as well as the absolute difference across arms. Log binomial regression will be used to calculate the relative risks of outcomes and corresponding 95% confidence intervals (CIs).

#### Sample size

Sample size was calculated based on the primary outcome of primary screening completion. These calculations relied on assumptions of clinical performance based on a systematic review of the relevant literature. We estimated that mailed self-sample HPV testing would be associated with a 12% improvement over standard recall for completion of primary screening [54, 55], with the proportion of participants who complete self-sampling ranging from 10 to 39% [56, 57]. To err conservatively and ensure adequate study power, we based our calculations on the assumption that 24% of screening non-attendees will complete mailed self-sample HPV testing compared to 18% anticipated to attend for clinic-based Pap testing after being recalled [45]. With 756 participants in each study arm and a total of 2268 participants in the trial (nQueryAdvisor version 7.0), we anticipate our ability to detect the indicated differences in proportions between any two groups. Calculations specified a two-sided alpha of 0.05.

### Data safety and monitoring

Trial safety is actively monitored by the Protocol Review and Monitoring Committee (PRMC) of the Dan L Duncan Comprehensive Cancer Center (Baylor College of Medicine). The PRMC monitors study progress and enrollment, adverse events, and data soundness. The trial was considered minimal risk by the reviewing IRB. Potential adverse events of self-sampling are comparable to those of routine clinic-based screening. Potential adverse events include pain, discomfort, and/or light bleeding. On the research information sheet included in the mailed box, participants are asked to self-report adverse events they experience by calling the study telephone number. All participant-reported adverse events will be documented in OnCore. Any serious adverse events will then be reported by the study team to the IRB. Research staff perform regular audits of OnCore and EMR data to ensure compliance with study procedures, notification and reporting of HR-HPV test results, and scheduling of clinical follow-up. No interim analyses are planned, nor are there pre-determine stopping rules based on a minimum threshold kit return rate.

## Discussion

Reducing disparities in screening coverage is critical for eliminating the global burden of cervical cancer [58]. As a health intervention, self-sample HPV testing offers promise for reducing screening disparities. Cervical self-sampling has been previously shown in numerous national and international studies to increase rates of participation in cervical cancer screening programs among otherwise underscreened women [13–23]. However, as with most evidence-based interventions, implementation requires thoughtful planning, deployment, and evaluation of the strategies used to support intervention adoption, delivery, and sustainability [59]. We hypothesize that patient-centered delivery systems, namely patient navigation, are important for increasing reach and response to the mailed intervention in safety net health systems. Telephone-based patient navigation provides a mechanism to motivate women and to address knowledge gaps and personal and cultural barriers to self-sampling [36]. Using a type 2 hybrid design, the PRESTIS trial will simultaneously evaluate the effectiveness of a mailed self-sample HPV testing as well as elucidate patient navigation as an implementation strategy to optimize its reach and response. Additionally, the trial will evaluate important implementation outcomes informed by the RE-AIM evaluation framework [41], including fidelity and adaptations made to the designed intervention. Surveys and in-depth interviews will assess acceptability and experiences among participants, a key consideration influencing the maintenance and sustainability of self-sample HPV testing as a usual care screening strategy for underscreened women. Finally, mathematical modeling will elucidate the cost-effectiveness of the intervention, a key consideration for health system decision-makers.

The main strength of PRESTIS lies in its pragmatic design, which allows for streamlined translation of research into practice. Using PRECIS-2 [43], pragmatic design decisions were incorporated along nine domains in the tool’s pragmatic trial framework. With regard to participant eligibility and recruitment, we established broad criteria to mirror criteria that would be used to determine eligibility for the interventions being studied were adopted into practice. To facilitate this goal, we sought and obtained a waiver of informed consent to identify and randomize eligible women from EMR data, as well as a waiver of documentation of informed consent for women to utilize the mailed kit (using a research information letter in lieu of a formal informed consent form). Substantial effort was made to integrate the delivery of the intervention into the healthcare system, including use of health system patient navigators to deliver the intervention, notify patients of results, and navigate them for clinical follow-up; using the health system laboratory for testing samples and processing results; and using usual care management of results follow-up. Clinical effectiveness outcomes will be analyzed using an intent-to-treat approach.

Despite major efforts to situate the trial in a real-world setting, certain design decisions should be considered when interpreting the trial’s results. Because safety net systems are used to varying extent by population, we instituted eligibility and exclusion criteria to minimize the inclusion of women who use the health system for emergency, inpatient, and specialty care but otherwise largely receive their primary care elsewhere. Specifically, we limited inclusion to women who had visited a primary care clinic at least twice over the past five years and excluded women who had a documented primary care provider outside of the health system. Our criteria for defining under-screened women included a six month “grace period” to allow for usual care interventions to be completed (i.e., flagging for past-due screening at primary care appointments, patient education) and activate women to obtain clinic-based screening. Thus, women only become eligible to participate in the trial once usual care activation interventions fail. While these criteria limit the number of Harris Health patients potentially eligible to participate in PRESTIS, it is likely that similar design decisions would be made if mailed self-sampling kits were implemented as a health system strategy. Two design decisions deviate from the original trial design. Direct return of kits to the laboratory was planned but later opted against due to the potential for misplacement of specimens coming into the high-volume, centralized laboratory. Kits instead are mailed to the patient navigation team, checked in, and then routed to the laboratory for testing. Direct posting of test results by the laboratory to the EMR was also planned but later opted against due to health system concerns over the posting of non-standard of care test results. Test results are instead documented as a research note. Finally, women’s decision to utilize the self-sampling kits may be influenced by the fact that they are delivered as part of a research study. We plan to elucidate the impact of this barrier in the participant surveys.

In conclusion, the broad purpose of the PRESTIS trial is to evaluate a pragmatic model for the integration of self-sample HPV testing in safety net health systems. To our knowledge, the trial is the first to be embedded in a U.S. safety net health system, an important setting considering that safety net health systems care for many of the medically underserved, racial/ethnic minority women [30] at greatest risk of cervical cancer [31, 33]. We expect trial findings to provide meaningful data to inform the equitable delivery of screening in order to realize the global goal of eliminating cervical cancer.

## Supplementary information

**Additional file 1.** SPIRIT checklist.

## Data Availability

Datasets generate during the study will be available to all involved researchers after completion of the trial. Data may be made available to outside researchers based on review of the request.

## References

[1] Gustafsson L, Pontén J, Zack M, Adami H-O (1997). International incidence rates of invasive cervical cancer after introduction of cytological screening. Cancer Causes Control.

[2] Chesson HW, Ekwueme DU, Saraiya M, Watson M, Lowy DR, Markowitz LE (2012). Estimates of the annual direct medical costs of the prevention and treatment of disease associated with human papillomavirus in the United States. Vaccine..

[3] White A, Thompson TD, White MC, Sabatino SA, de Moor J, Doria-Rose PV (2017). Cancer screening test use - United States, 2015. MMWR Morb Mortal Wkly Rep.

[4] Watson M, Benard V, King J, Crawford A, Saraiya M (2017). National assessment of HPV and Pap tests: changes in cervical cancer screening, national health interview survey. Prev Med.

[5] Howlader NNA, Krapcho M, Garshell J, Miller D, Altekruse SF, Kosary CL, Yu M, Ruhl J, Tatalovich Z, Mariotto A, Lewis DR, Chen HS, Feuer EJ, Cronin KA (2015). SEER cancer statistics review, 1975-2012.

[6] Janerich DT, Hadjimichael O, Schwartz PE, Lowell DM, Meigs JW, Merino MJ (1995). The screening histories of women with invasive cervical cancer, Connecticut. Am J Public Health.

[7] Benard VB, Thomas CC, King J, Massetti GM, Doria-Rose VP, Saraiya M (2014). Vital signs: cervical cancer incidence, mortality, and screening - United States, 2007-2012. MMWR Morb Mortal Wkly Rep.

[8] Goel MS, Wee CC, McCarthy EP, Davis RB, Ngo-Metzger Q, Phillips RS (2003). Racial and ethnic disparities in cancer screening: the importance of foreign birth as a barrier to care. J Gen Intern Med.

[9] Bosgraaf RP, Ketelaars PJW, Verhoef VMJ, Massuger LFAG, Meijer CJLM, Melchers WJG (2014). Reasons for non-attendance to cervical screening and preferences for HPV self-sampling in Dutch women. Prev Med.

[10] Crawford A, Benard V, King J, Thomas CC (2016). Understanding barriers to cervical cancer screening in women with access to care, behavioral risk factor surveillance system, 2014. Prev Chronic Dis.

[11] Ogunwale AN, Sangi-Haghpeykar H, Montealegre J, Cui Y, Jibaja-Weiss M, Anderson ML (2016). Non-utilization of the pap test among women with frequent health system contact. J Immigr Minor Health.

[12] Tota J, Ramana-Kumar A, El-Khatib Z, Franco E (2014). The road ahead for cervical cancer prevention and control. Curr Oncol.

[13] Gok M, Heideman DA, van Kemenade FJ, Berkhof J, Rozendaal L, Spruyt JW (2010). HPV testing on self collected cervicovaginal lavage specimens as screening method for women who do not attend cervical screening: cohort study. BMJ..

[14] Gok M, van Kemenade FJ, Heideman DA, Berkhof J, Rozendaal L, Spruyt JW (2012). Experience with high-risk human papillomavirus testing on vaginal brush-based self-samples of non-attendees of the cervical screening program. Int J Cancer.

[15] Giorgi Rossi P, Marsili LM, Camilloni L, Iossa A, Lattanzi A, Sani C (2011). The effect of self-sampled HPV testing on participation to cervical cancer screening in Italy: a randomised controlled trial (ISRCTN96071600). Br J Cancer.

[16] Sanner K, Wikstrom I, Strand A, Lindell M, Wilander E (2009). Self-sampling of the vaginal fluid at home combined with high-risk HPV testing. Br J Cancer.

[17] Wikstrom I, Lindell M, Sanner K, Wilander E (2011). Self-sampling and HPV testing or ordinary pap-smear in women not regularly attending screening: a randomised study. Br J Cancer.

[18] Piana L, Leandri FX, Le Retraite L, Heid P, Tamalet C, Sancho-Garnier H (2011). HPV-Hr detection by home self sampling in women not compliant with pap test for cervical cancer screening. Results of a pilot programme in Bouches-du-Rhone. Bull Cancer.

[19] Virtanen A, Nieminen P, Luostarinen T, Anttila A (2011). Self-sample HPV tests as an intervention for nonattendees of cervical cancer screening in Finland: a randomized trial. Cancer Epidemiol Biomark Prev.

[20] Bais AG, van Kemenade FJ, Berkhof J, Verheijen RH, Snijders PJ, Voorhorst F (2007). Human papillomavirus testing on self-sampled cervicovaginal brushes: an effective alternative to protect nonresponders in cervical screening programs. Int J Cancer.

[21] Tranberg M, Bech BH, Blaakaer J, Jensen JS, Svanholm H, Andersen B (2016). Study protocol of the CHOiCE trial: a three-armed, randomized, controlled trial of home-based HPV self-sampling for non-participants in an organized cervical cancer screening program. BMC Cancer.

[22] Broberg G, Gyrd-Hansen D, Miao Jonasson J, Ryd ML, Holtenman M, Milsom I (2014). Increasing participation in cervical cancer screening: offering a HPV self-test to long-term non-attendees as part of RACOMIP, a Swedish randomized controlled trial. Int J Cancer.

[23] Enerly E, Bonde J, Schee K, Pedersen H, Lönnberg S, Nygård M (2016). Self-sampling for human papillomavirus testing among non-attenders increases attendance to the Norwegian cervical cancer screening programme. PLoS One.

[24] Bouchard-Fortier G, Hajifathalian K, McKnight MD, Zacharias DG, Gonzalez-Gonzalez LA (2014). Co-testing for detection of high-grade cervical intraepithelial neoplasia and cancer compared with cytology alone: a meta-analysis of randomized controlled trials. J Public Health.

[25] Curry SJ, Krist AH, Owens DK, Barry MJ, Caughey AB, Davidson KW (2018). Screening for cervical cancer: US preventive services task force recommendation statement. JAMA..

[26] Ogilvie GS, Patrick DM, Schulzer M, Sellors JW, Petric M, Chambers K (2005). Diagnostic accuracy of self collected vaginal specimens for human papillomavirus compared to clinician collected human papillomavirus specimens: a meta-analysis. Sex Transm Infect.

[27] Petignat P, Faltin DL, Bruchim I, Tramer MR, Franco EL, Coutlee F (2007). Are self-collected samples comparable to physician-collected cervical specimens for human papillomavirus DNA testing? A systematic review and meta-analysis. Gynecol Oncol.

[28] Winer RL, Tiro JA, Miglioretti DL, Thayer C, Beatty T, Lin J (2018). Rationale and design of the HOME trial: a pragmatic randomized controlled trial of home-based human papillomavirus (HPV) self-sampling for increasing cervical cancer screening uptake and effectiveness in a U.S. healthcare system. Contemp Clin Trials.

[29] Lewin MEAS (2000). America’s health care safety net: intact but endangered.

[30] Chokshi DA, Chang JE, Wilson RM (2016). Health reform and the changing safety net in the United States. N Engl J Med.

[31] Watson M, Saraiya M, Benard V, Coughlin SS, Flowers L, Cokkinides V (2008). Burden of cervical cancer in the United States, 1998-2003. Cancer..

[32] Centers for Disease Control and Prevention (CDC) (2012). Human papillomavirus-associated cancers - United States, 2004-2008. MMWR Morb Mortal Wkly Rep.

[33] Yoo W, Kim S, Huh WK, Dilley S, Coughlin SS, Partridge EE (2017). Recent trends in racial and regional disparities in cervical cancer incidence and mortality in United States. PLoS One.

[34] Freeman HP (2006). Patient navigation: a community based strategy to reduce cancer disparities. J Urban Health.

[35] Baron RC, Rimer BK, Breslow RA, Coates RJ, Kerner J, Melillo S (2008). Client-directed interventions to increase community demand for breast, cervical, and colorectal cancer screening a systematic review. Am J Prev Med.

[36] Paskett ED, Harrop JP, Wells KJ (2011). Patient navigation: an update on the state of the science. CA Cancer J Clin.

[37] Curran GM, Bauer M, Mittman B, Pyne JM, Stetler C (2012). Effectiveness-implementation hybrid designs: combining elements of clinical effectiveness and implementation research to enhance public health impact. Med Care.

[38] Chambers DA, Glasgow RE, Stange KC (2013). The dynamic sustainability framework: addressing the paradox of sustainment amid ongoing change. Implement Sci.

[39] Landes SJ, McBain SA, Curran GM (2019). An introduction to effectiveness-implementation hybrid designs. Psychiatry Res.

[40] Aarons GA, Sklar M, Mustanski B, Benbow N, Brown CH (2017). “Scaling-out” evidence-based interventions to new populations or new health care delivery systems. Implement Sci.

[41] Damschroder LJ, Aron DC, Keith RE, Kirsh SR, Alexander JA, Lowery JC (2009). Fostering implementation of health services research findings into practice: a consolidated framework for advancing implementation science. Implement Sci.

[42] Green LW, Kreuter M, Deeds SG, Partridge KB (1980). Health education planning: a diagnostic approach.

[43] Loudon K, Treweek S, Sullivan F, Donnan P, Thorpe KE, Zwarenstein M (2015). The PRECIS-2 tool: designing trials that are fit for purpose. BMJ.

[44] Montealegre JR, Gossey JT, Anderson ML, Chenier RS, Chauca G, Rustveld LO (2014). Implementing targeted cervical cancer screening videos at the point of care. Patient Educ Couns.

[45] Massad LS, Einstein MH, Huh WK, Katki HA, Kinney WK, Schiffman M (2013). 2012 updated consensus guidelines for the management of abnormal cervical cancer screening tests and cancer precursors. Obstet Gynecol.

[46] Huh WK, Ault KA, Chelmow D, Davey DD, Goulart RA, Garcia FA (2015). Use of primary high-risk human papillomavirus testing for cervical cancer screening: interim clinical guidance. Obstet Gynecol.

[47] Harris PA, Taylor R, Thielke R, Payne J, Gonzalez N, Conde JG (2009). Research electronic data capture (REDCap)--a metadata-driven methodology and workflow process for providing translational research informatics support. J Biomed Inform.

[48] Harris PA, Taylor R, Minor BL, Elliott V, Fernandez M, O'Neal L (2019). The REDCap consortium: building an international community of software platform partners. J Biomed Inform.

[49] de Sanjose S, Quint WG, Alemany L, Geraets DT, Klaustermeier JE, Lloveras B (2010). Human papillomavirus genotype attribution in invasive cervical cancer: a retrospective cross-sectional worldwide study. Lancet Oncol.

[50] Nghiem VT, Davies KR, Beck JR, Follen M, Cantor SB (2016). Overtreatment and cost-effectiveness of the see-and-treat strategy for managing cervical precancer. Cancer Epidemiol Biomark Prev.

[51] Rozemeijer K, de Kok IM, Naber SK, van Kemenade FJ, Penning C, van Rosmalen J (2015). Offering self-sampling to non-attendees of organized primary HPV screening: when do harms outweigh the benefits?. Cancer Epidemiol Biomark Prev.

[52] McCrory DC, Matchar DB, Bastian L, Datta S, Hasselbad V, Hickey J, et al. Evaluation of Cervical Cytology: Summary. 1999. In: AHRQ Evidence Report Summaries. Rockville (MD): Agency for Healthcare Research and Quality (US); 1998-2005. Available from: https://www.ncbi.nlm.nih.gov/books/NBK11840/. PMC4781480 11925972

[53] Myers ER, McCrory DC, Nanda K, Bastian L, Matchar DB (2000). Mathematical model for the natural history of human papillomavirus infection and cervical carcinogenesis. Am J Epidemiol.

[54] Cuzick J, Clavel C, Petry KU, Meijer CJ, Hoyer H, Ratnam S (2006). Overview of the European and North American studies on HPV testing in primary cervical cancer screening. Int J Cancer.

[55] Verdoodt F, Jentschke M, Hillemanns P, Racey CS, Snijders PJ, Arbyn M (2015). Reaching women who do not participate in the regular cervical cancer screening programme by offering self-sampling kits: a systematic review and meta-analysis of randomised trials. Eur J Cancer.

[56] Brouwers MC, De Vito C, Bahirathan L, Carol A, Carroll JC, Cotterchio M (2011). What implementation interventions increase cancer screening rates? A systematic review. Implement Sci.

[57] Szarewski A, Cadman L, Mesher D, Austin J, Ashdown-Barr L, Edwards R (2011). HPV self-sampling as an alternative strategy in non-attenders for cervical screening - a randomised controlled trial. Br J Cancer.

[58] Ghebreyesus TA (2019). Global strategy towards eliminating cervical cancer as a public health problem.

[59] Proctor EK, Powell BJ, McMillen JC (2013). Implementation strategies: recommendations for specifying and reporting. Implement Sci.

[60] International Committee of Medican Journal Editors. Roles and Responsibilities of Authors, Contributors, Reviewers, Editors, Publishers, and Owners. Available at https://www.icmje.org/recommendations/browse/roles-and-responsibilities. Accessed 17 Sept 2020.

